# Semireplication-competent vesicular stomatitis virus as a novel platform for oncolytic virotherapy

**DOI:** 10.1007/s00109-012-0863-6

**Published:** 2012-01-28

**Authors:** Alexander Muik, Catherine Dold, Yvonne Geiß, Andreas Volk, Marina Werbizki, Ursula Dietrich, Dorothee von Laer

**Affiliations:** 1 Georg-Speyer-Haus, 60596 Frankfurt am Main, Germany; 2 Institute for Virology, Innsbruck Medical University, Fritz-Pregl-Str. 3, A-6020 Innsbruck, Austria

**Keywords:** Vesicular stomatitis virus, Oncolytic virus, Virotherapy, Malignant glioma

## Abstract

Among oncolytic viruses, the vesicular stomatitis virus (VSV) is especially potent and a highly promising agent for the treatment of cancer. But, even though effective against multiple tumor entities in preclinical animal models, replication-competent VSV exhibits inherent neurovirulence, which has so far hindered clinical development. To overcome this limitation, replication-defective VSV vectors for cancer gene therapy have been tested and proven to be safe. However, gene delivery was inefficient and only minor antitumor efficacy was observed. Here, we present semireplication-competent vector systems for VSV (srVSV), composed of two *trans*-complementing, propagation-deficient VSV vectors. The de novo generated deletion mutants of the two VSV polymerase proteins P (phosphoprotein) and L (large catalytic subunit), VSVΔP and VSVΔL respectively, were used mutually or in combination with VSVΔG vectors. These srVSV systems copropagated in vitro and in vivo without recombinatory reversion to replication-competent virus. The srVSV systems were highly lytic for human glioblastoma cell lines, spheroids, and subcutaneous xenografts. Especially the combination of VSVΔG/VSVΔL vectors was as potent as wild-type VSV (VSV-WT) in vitro and induced long-term tumor regression in vivo without any associated adverse effects. In contrast, 90% of VSV-WT-treated animals succumbed to neurological disease shortly after tumor clearance. Most importantly, even when injected into the brain, VSVΔG/VSVΔL did not show any neurotoxicity. In conclusion, srVSV is a promising platform for virotherapeutic approaches and also for VSV-based vector vaccines, combining improved safety with an increased coding capacity for therapeutic transgenes, potentially allowing for multipronged approaches.

**Electronic supplementary material:**

The online version of this article (doi:10.1007/s00109-012-0863-6) contains supplementary material, which is available to authorized users.

## Introduction

The use of viruses as targeted cancer therapeutics has shown significant promise in the last few years. Especially the vesicular stomatitis virus (VSV), a relatively new player in the oncolytic virotherapy field, has proven to be effective against a variety of tumor entities such as malignant glioma [1, 2], hepatocellular carcinoma [3, 4], prostate cancer [5, 6], and ovarian carcinoma [7]. However, to date, the inherent neurotoxicity of VSV has hindered clinical development since intracerebral administration causes fatal encephalitis in rodents and nonhuman primates [8, 9]. Thus, replication-competent VSV is associated with an increased risk of systemic dissemination and potentially severe pathology if it enters the CNS. Therefore, attenuated virus variants and propagation-deficient viral vectors were generated. Unfortunately, the reduced toxicity of attenuated replication-competent VSV is invariably accompanied with some reduction of replicative and oncolytic activity [10, 11], whereas the major limitation of propagation-deficient viral vectors has been the inefficient transduction rate of cancer cells in vivo [2, 12].

A new strategy to potentially enhance safety of replication-competent VSV while increasing the capacity for therapeutic transgenes is the use of a semireplication-competent vector system similar to those described for retroviruses and adenoviruses [13, 14]. Here, we successfully developed a semireplication-competent vector system for VSV (srVSV), which is based on two *trans*-complementing propagation-deficient VSV vectors. The genes essential for viral replication are divided onto two separate packageable vector genomes, so that infectious progeny can only be produced in double-infected host cells. Importantly, the VSV RNA genome does not undergo genetic reassortment or recombination, making it unlikely that the binary system reverts into a replication-competent recombinant VSV [15]. In this study, we used the propagation-deficient, eGFP-expressing VSV*ΔG-vector [16], which lacks the G gene, in combination with de novo synthesized and rescued deletion mutants VSVΔP-DsRed and VSVΔL-DsRed, lacking the genes P and L, respectively, that encode the components of the viral polymerase complex. Accordingly, three different srVSV combinations were feasible: VSV*ΔG/VSVΔP-DsRed (srVSV(ΔG/ΔP)), VSV*ΔG/VSVΔL-DsRed (srVSV(ΔG/ΔL)), and VSVΔP-DsRed/VSVΔL-DsRed (srVSV(ΔP/ΔL)). All srVSV systems allowed for in vitro reciprocal complementation thus leading to copropagation associated with clear antitumor potency against human glioblastoma cell lines. In addition, the most potent vector combination, srVSV(ΔG/ΔL), was tested in a preclinical subcutaneous (s.c.) glioblastoma mouse model and proved to be only slightly attenuated compared to wild-type VSV (VSV-WT). Tumors regressed in both cohorts, but in contrast to the srVSV-treated group, 90% of VSV-WT-treated animals succumbed to viral neurotoxicity. Most importantly, neither srVSV treatment of tumor-bearing animals nor direct intracranial administration in healthy mice was associated with any sign of neurotoxicity. Eventually, all srVSV systems proved to be safe as we have not been able to detect any sign of recombinatory reversion to the wild-type strain.

## Materials and methods

### Cell culture

BHK-21 baby hamster kidney and U-87 MG human glioblastoma cells were obtained from the American Type Culture Collection (Manassas, VA). G62 human glioblastoma cells were kindly provided by M. Westphal (University Hospital Eppendorf, Hamburg, Germany). HEK 293-NPeGFPL (clone 206) stably expressing VSV-N, P, and L protein were a gift from A. Pattnaik (University of Nebraska, Lincoln, USA) [17]. All cells were kept in a humidified atmosphere containing 5% CO_2_ at 37°C. BHK-21, U-87 MG, G62, and 293-NPeGFPL cells were maintained in DMEM (Gibco) supplemented with 10% FBS (Perbio Science). 293-NPeGFPL cells were kept under G418 selection.

### Viruses

**Fig. 1:**
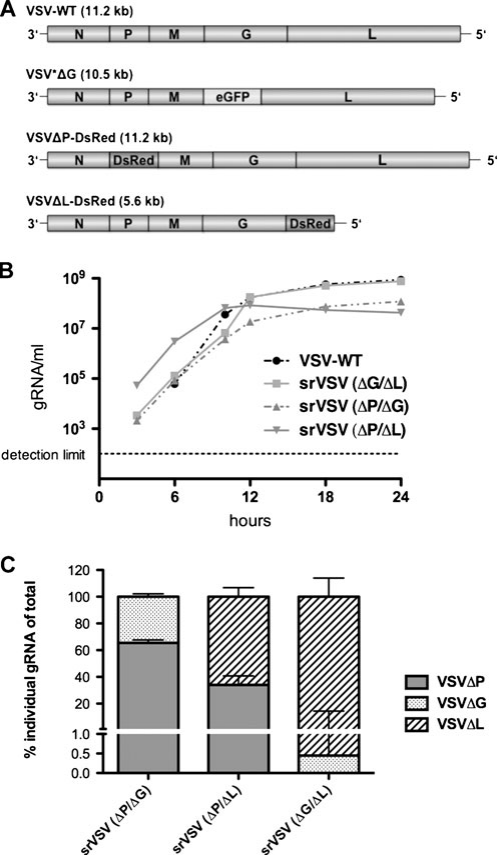
Construction and functional characterization of srVSV systems. **a** Schematic representation of the recombinant vesicular stomatitis virus (*VSV*) genomes. Genomes and the respective open reading frames are presented in 3′-5′ orientation. **b** Multicycle growth curves of srVSV systems compared to VSV-WT. BHK-21 cells were infected with VSV-WT or the respective srVSV systems at an MOI of 0.05. At the indicated time points postinfection, culture supernatants were collected and viral genomic RNA was determined by real-time RT-PCR. Virus titers of *n* = 2 infection experiments are shown as mean±SD. **c** Symmetry of individual vector genome contributions during copropagation was assessed via real-time RT-PCR using two independent primer/probe sets. Ratios of individual vector titers per total vector concentration are shown as mean±SD

The propagation-incompetent VSV*ΔG vector, coding for eGFP as reporter, as well as the particularly strong type I interferon (IFN) inducing VSV*M_Q_, a replication-competent VSV with multimutated matrix protein (VSV-M), have been described previously [16, 18]. The deletion mutants VSVΔP-DsRed and VSVΔL-DsRed were generated de novo: To exchange the VSV-P gene for DsRed, the N-P intergenic region (IGR) and a part of the VSV-N gene as well as the P-M IGR and a part of the M gene were PCR amplified from pVSV-XN2 using the primers 5′-CGAT**CTCGAG**GTATACATCTCTTACTACAGCAGG-3′/5′-CAGT**GAATTC**GATATCTGTTAGTTTTTTTCATATGTAGC-3′ (N-P IGR) and 5′-CGAT**GCGGCCGC**ACTATGAAAAAAAGTAACAGATATCACG-3′/5′-CAGT**CCGCGG**ACGCGTAAACAGATCGATCTCTG-3′ (P-M IGR) with unique restriction sites (shown in bold). In parallel, DsRed was subcloned from pDsRed-Express-N1 (Clontech) into the multiple cloning site (MCS) of the pBluescript-II cloning vector (Stratagene) with *Bam*HI/*Not*I. Subsequently, PCR products were digested with *Xho*I/*Eco*RI (N-P IGR) and *Not*I/*Sac*II (P-M IGR) and sequentially cloned in front and behind the DsRed gene. Finally, the DsRed cassette was excised with *Bst*Z17i/*Mlu*I and inserted into the *Bst*Z17i/*Mlu*I site of pVSV-XN2, replacing VSV-P to yield pVSVΔP-DsRed. A similar cloning strategy was applied to generate pVSVΔL-DsRed: The G-L IGR and a part of the VSV-G gene as well as the L-HDV ribozyme region were PCR amplified from pVSV-XN2 using the primers 5′-CAGT**GGTACC**CTAAAATACTTTGAGACCAG-3′/5′-CGAT**GGATCC**GATTGCTGTTAGTTTTTTTCATAAAAATTAAAAACTC-3′ (G-L IGR) and 5′-CAGT**GCGGCCGC**AAAATCATGAGGAGACTCCAAACTTTAAG-3′/5′-CGAT**GAGCTC**GCACTAGTATCGAGGTCTCGATC-3′ (L-HDV ribozyme) with unique restriction sites (shown in bold). PCR products were digested with *Kpn*I/*Bam*HI (G-L IGR) and *Not*I/*Sac*I (L-HDV ribozyme) and cloned in front and behind the DsRed gene in the pBluescript-II-DsRed vector. Finally, DsRed was excised with *Nhe*I/*Spe*I and inserted into the *Nhe*I/*Spe*I site of pVSV-XN2, yielding pVSVΔL-DsRed. Novel recombinant viruses (Fig. 1a) were rescued as described previously [19]. To produce infectious virions, VSV*ΔG-vectors were propagated on BHK-21 cells transiently expressing VSV-G [20]. VSVΔP-DsRed and VSVΔL-DsRed were amplified on 293-NPeGFPL cells. Vector titers were determined as 50% tissue culture infective dose (TCID_50_) using the Spearman–Kärber method [21]. VSVΔP-DsRed and VSVΔL-DsRed titration was performed on 293-NPeGFPL cells, VSV-WT and VSV*M_Q_ were titrated on BHK-21 cells, and VSV*ΔG titration was performed on BHK-GP [20].

### Quantitative PCR-based multicycle growth curve analysis

BHK-21 cells were infected in 6-well plates (10^6^ cells/well) with a multiplicity of infection (MOI) of 0.05 of each individual vector of the three potential srVSV vector systems or VSV-WT as positive control. Filtered (0.45 μm) supernatants were collected at the indicated time points, and RNA was extracted from 50 μl supernatant using the RNeasy Mini Kit (Qiagen). RNA was reverse transcribed using the High Capacity RNA-to-cDNA Kit (Applied Biosystems). Vector propagation was monitored via real-time RT-PCR to determine the total VSV genomic RNA (gRNA) amount in supernatants [19]. Known plasmid amounts were used to determine the standard curve for real-time RNA quantification. Two independent qPCR primer and probe sets were used, spanning the N-P and the M-G IGR of the VSV genome (see Supplementary Fig. S1c, d). Real-time PCR was carried out with the TaqMan® Gene Expression Master Mix (Applied Biosystems) using a LightCycler® 480 Real-Time PCR System (Roche). For both applied real-time PCRs, the detection limit was 10^2^ gRNA/ml.

### In vitro cytotoxicity assay

Human glioblastoma cells were plated in 96-well plates at 10^4^ cells/well in 100 μl medium. Cells were cultured as monolayer or multicellular tumor spheroids. For spheroid cultures, 96-well plates were precoated with 75 μl 1% agar noble (Difco). Cultures were infected with the respective viral system (srVSV or VSV-WT) at an MOI of 0.2 or treated with phosphate-buffered saline (PBS) the following day. Cell viability was assayed in dodecaplicates in *n* = 3 independent experiments at the indicated time points postinfection using the cell proliferation agent WST-1 (Roche). Results are expressed as percentage of viable cells compared to PBS-treated controls.

### Animal studies

For antitumor efficacy testing, 6-week old NOD/SCID mice (Jackson Laboratories) were anesthetized with isoflurane and 10^6^ G62 human glioblastoma cells were subcutaneously injected into the left and right flanks. Tumor growth was monitored with a caliper. At a tumor volume of 0.1 cm^3^, mice were treated intratumorally with two doses of either 2.8 × 10^5^ TCID_50_ srVSV(ΔG/ΔL) or 2.8 × 10^5^ TCID_50_ VSV-WT and PBS as controls. Bilateral tumors were treated alike. When tumor size exceeded 0.8 cm^3^, mice were sacrificed. In addition, two mice were sacrificed at 3 days post-srVSV treatment and s.c. tumors were prepared for immunofluorescence analysis.

For neurotoxicity analysis, 6-week old CD1 Swiss mice (Charles River) were anesthetized by intraperitoneal injection of ketamine/xylazine (100 and 10 mg/kg of body weight, respectively). 10^2^, 10^3^, and 10^4^ TCID_50_ srVSV(ΔG/ΔL), as well as 1.4 × 10^1^ and 1.4 × 10^4^ TCID_50_ VSV-WT or PBS were stereotactically injected into the right frontal lobe of mice brains (1.5 mm lateral, 2 mm rostral to the bregma at 2 mm depth). Animals were monitored for signs of neurological impairment. Two mice of the 10^4^ TCID_50_ srVSV(ΔG/ΔL)-treated group were sacrificed at 3 days postinjection (dpi), and brains were prepared for immunofluorescence analysis. The brains were sectioned (40 μm) on a Leica VT1000S vibratome (Leica, Bensheim, Germany). Nuclear counterstaining was performed with TO-PRO-3 iodide (Invitrogen). Sections were analyzed by confocal laser scanning microscopy using a Nikon C1S1 microscope (Nikon, Düsseldorf, Germany). All procedures were approved by the governmental board for the care of animal subjects (Regierungspräsidium Darmstadt, Germany).

### Stimulation and IFN-α detection

Murine bone marrow (BM)-derived plasmacytoid dendritic cells (pDCs) were generated as previously described [22]. In brief, BM cells were flushed from femur and tibia with RPMI supplemented with 10% FBS (Perbio Science). Erythrocytes were lysed, cells were washed, and single-cell suspensions were cultivated for 8 days in medium supplemented with 100 ng/ml Flt3-L (R&D Systems). As determined by FACS analysis, Flt3-L cultures consisted of ≈20% CD11c^+^B220^+^ pDCs (data not shown). For IFN stimulation experiments, 2 × 10^6^ Flt3-L-stimulated BM-pDC bulk culture cells were seeded per 24 well. Cultures were infected with either srVSV, VSV*ΔG, VSVΔL-DsRed, VSV-WT, or VSV*M_Q_ (each *n* = 2) at an MOI of 2. Supernatants were collected at 24 h postinfection (hpi) and analyzed for IFN-α via ELISA (PBL Biomedical Laboratories).

### Statistical analysis

For comparison of individual time points or columns, statistical difference was determined using unpaired *t* test. Mice survival curves were plotted as Kaplan–Meier analysis, and statistical significance between treatment groups was compared using the log-rank test.

## Results

Novel recombinant viruses were cloned based on the pVSV-XN2 plasmid background and rescued as described previously [19]. A schematic representation of the VSV vector genomes is shown in Fig. 1a, and their identity was confirmed by gene-specific RT-PCR (Supplementary Fig. S1a, b). Both deletion mutants, VSVΔP-DsRed and VSVΔL-DsRed, were unable to propagate and did not generate progeny virions in cell cultures not providing the respective deleted viral gene in trans, as real-time RT-PCR (Supplementary Fig. S1c, d) of supernatants were negative for VSV gRNA (data not shown).

### srVSV(ΔG/ΔL) is the most potent srVSV system in terms of vector propagation

In order to assess the replication competence of the three potential srVSV systems, BHK-21 cells were infected with an MOI of 0.05 of each individual vector or VSV-WT as control to generate multicycle growth curves. Vector propagation was monitored on the gRNA level via real-time RT-PCR [19]. In VSV-WT-infected cultures, gRNA associated with secreted progeny virions was first detectable at 6 hpi, reaching a plateau around 12–18 hpi with maximum titers of more than 8 × 10^8^ gRNA/ml (8.77 × 10^8^ ± 9.28 × 10^7^ gRNA/ml, see Fig. 1b). In comparison, all srVSV vector systems showed an earlier onset of replication with first gRNA detectable at 3 hpi and srVSV(ΔP/ΔL) being the most potent in the initial phase with titers of 5.33 × 10^4^ ± 3.05 × 10^3^ gRNA/ml 3 hpi. Both, the srVSV(ΔP/ΔG) and the srVSV(ΔG/ΔL) system lagged behind with titers being about tenfold reduced 3–6 hpi. Consistently, srVSV(ΔP/ΔL) was also the first to reach its plateau at 10–12 hpi with a maximum of 8.44 × 10^7^ ± 3.63 × 10^6^ gRNA/ml before its titer slowly started to regress. In contrast, both srVSV(ΔP/ΔG) and srVSV(ΔG/ΔL) ended up with a more robust replication, reaching titers of 1.19 × 10^8^ ± 1.63 × 10^6^ gRNA/ml for srVSV(ΔP/ΔG) and 7.60 × 10^8^ ± 4.47 × 10^7^ gRNA/ml for srVSV(ΔG/ΔL) at 24 hpi. Thus, the binary system using VSV*ΔG and VSVΔL-DsRed was the most potent srVSV system in terms of vector dissemination even reaching maximum gRNA titers comparable to VSV-WT.

In parallel, srVSV functional titers of supernatants collected at 24 hpi were determined as TCID_50_ per milliliter, as double-infected cells are a prerequisite to initiate copropagation. Correspondingly, the TCID_50_ of srVSV systems were 180- (srVSV(ΔG/ΔL)) to 2,000-fold (srVSV(ΔP/ΔL)) lower than their gRNA titers, primarily reflecting the chance of coinfection, and to a considerably lesser extent reflecting the difference between genome and functional titers, as VSV-WT gRNA titers were only 6-fold higher compared to the respective TCID_50_. However, consistent with the maximum obtained VSV gRNA per milliliter concentrations during the multicycle growth curve, srVSV(ΔG/ΔL) displayed the highest TCID_50_ per milliliter of 4.22 × 10^6^, whereas titers for srVSV(ΔP/ΔG) were approx. 20-fold and for srVSV(ΔP/ΔL) around 100-fold lower (Table 1).

**Table 1:** Functional titers

Viral system	TCID_50_/ml
VSV-WT	1.58 × 10^8^
srVSV(ΔG/ΔL)	4.22 × 10^6^
srVSV(ΔP/ΔG)	2.37 × 10^5^
srVSV(ΔP/ΔL)	4.22 × 10^4^

### srVSV systems are characterized by asymmetric copropagation

As srVSV systems are composed of two vectors with different properties such as gene composition and genome size, the mode of copropagation during the multicycle growth curve was analyzed via two independent qPCRs with amplicons spanning the N-P or M-G IGR of the VSV genome (Supplementary Fig. S1c, d). Combining the obtained qPCR data, single-vector titers were calculated as ratio of the individual vector gRNA per milliliter per total vector gRNA per milliliter for all time points of the multicycle growth curve. As ratios proved to be consistent for each srVSV system throughout the whole observation period of 24 h, time-independent means and standard deviations were calculated. Indeed, the assessment revealed that vector copropagation was not due to symmetric replication of both vector genomes, but could rather be characterized as an asymmetric process (Fig. 1c). Each srVSV system could be defined by a distinct preference of one vector over the other. In case of srVSV(ΔP/ΔG), the VSVΔP vector accounts for 65.49±2.16% and VSVΔG for 34.51 ± 2.16% of the total titer. Even more pronounced is the asymmetry in favor of the VSVΔL vector with a share of 65.99 ± 6.78% (ΔP/ΔL) and 99.55 ± 14.03% (ΔG/ΔL) of the total progeny generated.

### Time lapse fluorescence microscopy of srVSV(ΔG/ΔL) copropagation

**Fig. 2:**
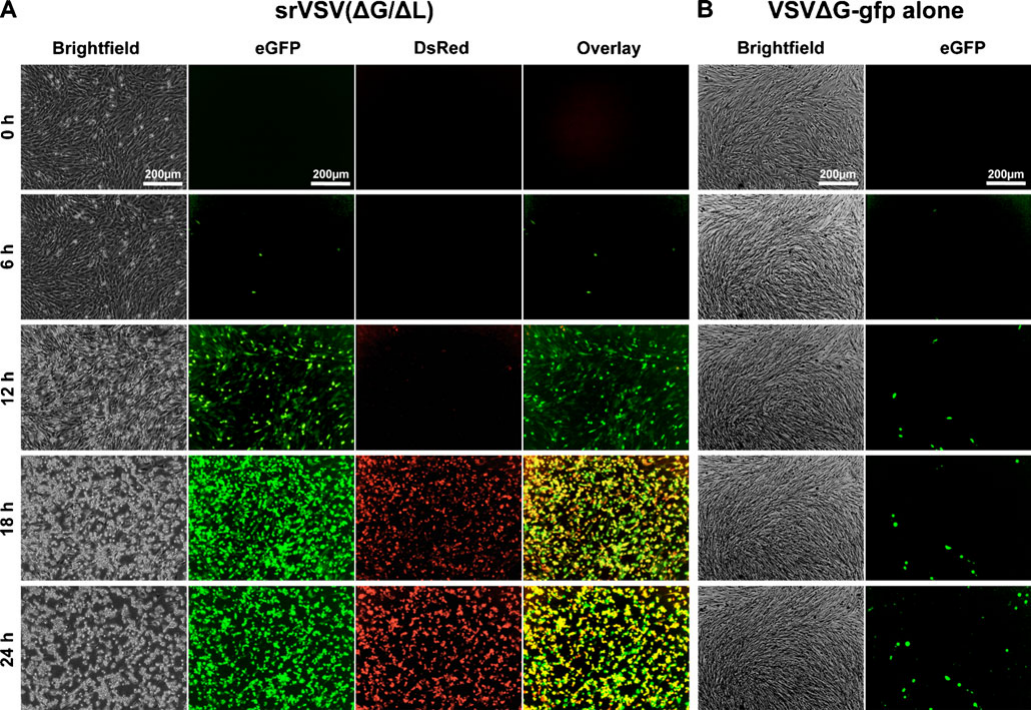
Copropagation of srVSV(ΔG/ΔL) in vitro. BHK-21 cells were infected with either **a** both defective VSV vectors or **b** only VSV*ΔG at an MOI of 0.05 and monitored by fluorescence time lapse microscopy for 24 h. Representative micrographs taken at the indicated time points are shown (time lapse movies are published as Supplementary Videos S1 and S2)

In a separate experiment, srVSV(ΔG/ΔL) copropagation was monitored by fluorescence time lapse microscopy over a period of 24 h after infecting BHK-21 cells at an MOI of 0.05 (Supplementary Video S1). As negative control, BHK-21 cells were infected with VSV*ΔG only. Representative micrographs at 0, 6, 12, 18, and 24 hpi are shown in Fig. 2. First eGFP fluorescence was detectable at 6 hpi, whereas both, DsRed-fluorescence and cytopathic effects (CPE), were confined to the srVSV-treated culture and were not detectable prior to 12 hpi (Fig. 2a). The dissemination of the srVSV system could be tracked by a gradual increase in eGFP^+^/DsRed^+^ cells as well as progressing CPE reaching a maximum at 24 hpi. In contrast, single-vector infected cultures did not show any propagation with only single-infected eGFP^+^ cells detectable throughout the observation period (see Fig. 2b and Supplementary Video S2).

### srVSV shows no sign of recombinatory reversion to replication competence

**Fig. 3:**
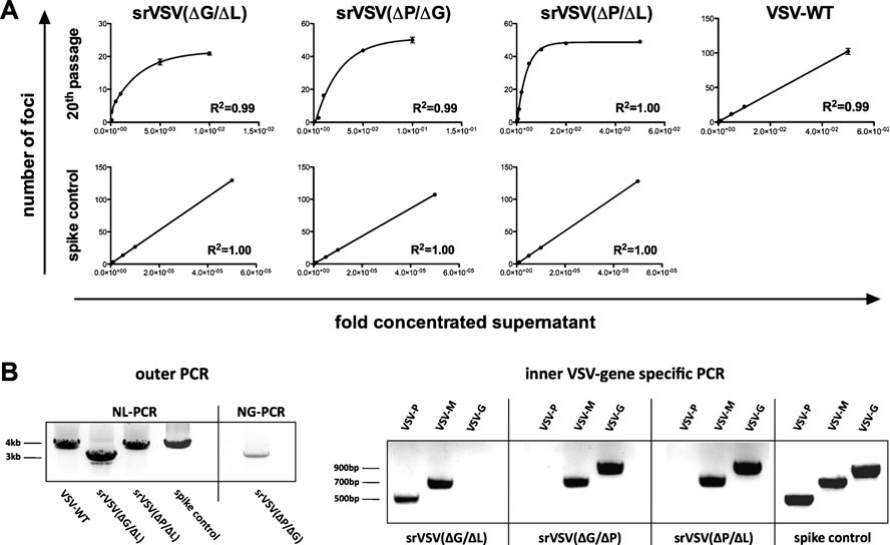
srVSV systems did not revert to full replication competence after 20 consecutive passages at limiting dilutions. srVSV vector pairs were subjected to repeated passaging (20 passages) on BHK-21 cells at limiting dilutions. As positive control, srVSV systems were spiked with ten TCID_50_ VSV-WT. At passage 20, serial dilutions of culture supernatants were tested for recombinants via plaque assay and viral RNA was isolated and reverse transcribed. **a** Supernatant concentration dependence of the number of formed foci. Serial dilutions of supernatants were tested in triplicates (*n* = 3) and number of foci is shown as mean±SD. Data points were fitted with either linear regression for VSV-WT and spike controls or nonlinear regression (biphasic decay) for srVSV systems. **b** Direct testing for potential recombination was performed via VSV gene deletion specific analytical nested PCR. Agarose gel electrophoresis of the obtained amplicons is shown

The main idea to develop srVSV for oncolytic virotherapy is to increase the integral safety compared to the replication-competent counterpart. Accordingly, genomic stability is an absolute requirement so that a recombinatory reversion of the binary system, potentially restoring full replicative capacity, can be precluded. In order to study viral genome stability, srVSV systems were subjected to end point dilution passage on BHK-21 cells to enable enrichment of potential functional revertants analogous to Taucher et al. [23]. To test this experimental setup, the srVSV systems were spiked with ten TCID_50_ VSV-WT as internal control to see whether the replication-competent virus can selectively be enriched. Already after passage 2 for srVSV(ΔP/ΔL), passage 3 for srVSV(ΔP/ΔG), and passage 4 at limiting dilutions for srVSV(ΔG/ΔL), the control-treated BHK-21 cells showed virus-induced CPE at low supernatant concentrations (down to 10^−7^) without any fluorescence detectable. In comparison, the unspiked srVSV vector systems copropagated only at high supernatant concentrations (down to 10^−4^), whereas only single-positive cells were found in cultures treated with low concentrations (down to 10^−6^). After 20 consecutive passages, serial dilutions of srVSV supernatants were tested for the number of focus-forming units via plaque assay on BHK-21 cells. All srVSV systems formed eGFP^+^/DsRed^+^ foci at high supernatant concentrations (down to 10^−4^) with its number not being directly proportional to the respective concentration. Instead, the interdependency between supernatant concentration and focus number could be characterized by a nonlinear biphasic decay with a goodness of fit of *R* ^2^ = 0.99 (ΔG/ΔL), *R* ^2^ = 0.99 (ΔP/ΔG), and *R* ^2^ = 1.00 (ΔP/ΔL, Fig. 3a), respectively. This is in absolute accordance with two individual replication-defective viral vectors constituting the copropagation-initializing unit. In comparison, the interdependency for the respective spiking controls as well as VSV-WT could be fitted by linear regression (*R* ^2^ in between 0.99 and 1.0), as would be expected for a replication-competent virus.

Next, we also looked directly for potential recombination events by RT-PCR as a more sensitive means. cDNA of the 20th passage of the srVSV systems and the respective spiking controls was prepared and used to perform a nested PCR. The outer PCR selectively amplified the genome of one recombinant vector out of the binary system as the reverse primer binding site is constituted in the gene deletion of the other vector genome (Supplementary Fig. S2). For srVSV(ΔG/ΔL) the VSV*ΔG genome was amplified, whereas for srVSV(ΔP/ΔG) and srVSV(ΔP/ΔL) the VSVΔP-DsRed genome was amplified. The inner VSV gene-specific PCRs then allowed us to check for potential recombination events at the locus of the actual gene deletion. Consistent with the phenotypic analysis (Fig. 3a), we have not been able to detect any recombination event for the srVSV systems. The VSV-G (for srVSV(ΔG/ΔL)) and VSV-P gene (for srVSV(ΔP/ΔG) and srVSV(ΔP/ΔL)) were not detectable, whereas the according amplicons were detected for the spiking control (see Fig. 3b). These data were corroborated by sequence analysis of the outer PCR amplicons, which clearly evidenced presence of the respective fluorescence marker gene (data not shown). Thus, in both, the phenotypic and genotypic analysis, recombination among the vector genomes was not detectable while the respective spiking controls were positive, the latter validating the applicability of the applied assays.

### srVSV exhibits antitumor activity in vitro and in vivo

**Fig. 4:**
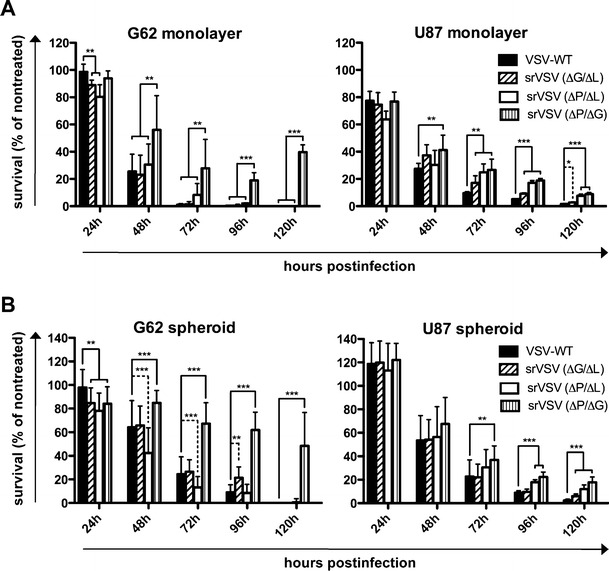
srVSV is comparable to VSV-WT with regard to its in vitro antitumor efficacy. **a** G62 and U87 human glioblastoma cells were infected with srVSV or VSV-WT at an MOI of 0.2. Cell viability was assayed at the indicated time points via WST-1 assay compared to untreated controls. **b** G62 and U87 human glioblastoma cells were grown as multicellular spheroids on agar. After spheroids had formed, cultures were infected with srVSV or VSV-WT at an MOI of 0.2. Cell viability was assayed at the indicated time points via WST-1 assay compared to untreated controls. Bars show mean±SD of three independent experiments (*n* = 3) performed in dodecaplicates. *p < 0.05, **p < 0.01, ***p < 0.001

Since the srVSV systems are to be used therapeutically as anticancer agents, we assessed its antitumor potency against two different human glioblastoma cell lines, G62 and U87, in vitro as well as in a s.c. G62 xenograft model in vivo. G62 and U87 cells were infected at an MOI of 0.2 with the respective viral system, and cell viability was monitored compared to untreated controls using the WST-1 assay. In addition to monolayer cultures (Fig. 4a), multicellular spheroids of both cell lines were also used (Fig. 4b), as spheroids represent an appropriate in vitro simulation of solid three-dimensional tumors resembling some of its regional heterogeneity also found in vivo [24, 25]. In the initial phase at 24 hpi, no significant differences in cell viability could be observed for U87 cells treated with the different viral systems. On the other hand, srVSV-treated G62 cultures showed significant differences compared to VSV-WT (98.69 ± 5.59% survival) initially at 24 hpi: G62 monolayers were reduced in cell viability for srVSV(ΔG/ΔL) (88.97 ± 3.55% survival) and srVSV (ΔP/ΔL) (80.25±8.87% survival, both p<0.01; Fig. 4a), whereas G62 spheroids showed increased cytopathic effects for all three srVSV systems tested relative to VSV-WT (*p* < 0.01, Fig. 4b). At all other time points (48–120 hpi), infected glioma cultures showed gradual reduction of cell viability due to viral CPE. For G62 monolayer cultures, srVSV(ΔG/ΔL) and srVSV(ΔP/ΔL) were as potent as VSV-WT. In contrast, srVSV(ΔP/ΔG) showed significantly reduced antitumor efficacy compared to all other viral systems (*p* < 0.001). Similar results were obtained for G62 spheroid cultures, with srVSV(ΔP/ΔG) lagging behind in its cytotoxicity (*p* < 0.001) and srVSV(ΔP/ΔL) being the most potent closely followed by srVSV(ΔG/ΔL). For U87 cultures, differences in cell viability between different treatment groups were not as strong: srVSV(ΔP/ΔG)-treated cultures were clearly the most viable (ranging from *p* < 0.01 to *p* < 0.001), whereas the srVSV(ΔG/ΔL) system performed best with regard to its antitumor effect. However, all srVSV-treated groups showed attenuated antitumor efficacy compared to VSV-WT-treated cultures.

**Fig. 5:**
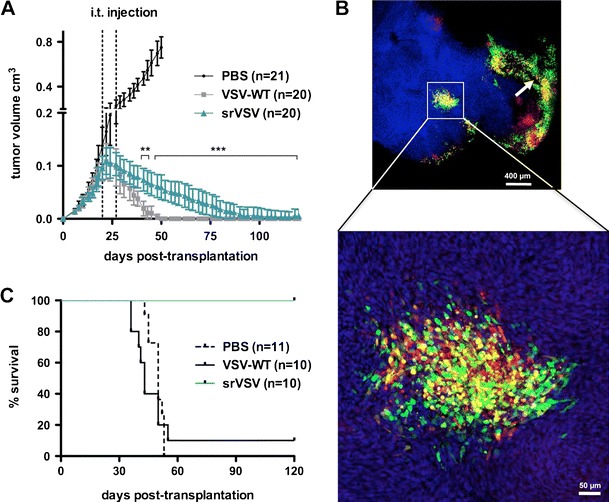
srVSV shows copropagation in vivo leading to antitumor activity without adverse effects. NOD/SCID mice were subcutaneously transplanted with 10^6^ G62 human glioblastoma cells per flank. At a tumor size of approximately 0.1 cm^3^, tumors were either treated with PBS, 2.8 × 10^5^ TCID_50_ VSV-WT or 2.8 × 10^5^ TCID_50_ srVSV(ΔG/ΔL). **a** Tumor growth was assessed with a caliper. Mice with tumors exceeding 0.8 cm^3^ were sacrificed. **b** Two representative srVSV-treated mice were sacrificed at 3 dpi, tumors were removed and suspended in PBS/4% paraformaldehyde. Tumor sections (40 μm) were prepared and nuclear counterstaining was performed with TO-PRO-3 iodide. **c** Kaplan–Meier survival analysis of the respective treatment groups. **p < 0.01, ***p < 0.001. *i.t.* intratumoral, *arrow* injection site

To determine whether srVSV is also effective in vivo, the most promising srVSV system, srVSV(ΔG/ΔL), was directly injected into s.c. bilateral G62 tumor xenografts at 2.8 × 10^5^ TCID_50_. Control tumors were either injected with PBS or 2.8 × 10^5^ TCID_50_ VSV-WT. PBS-treated tumors grew rapidly, and all mice had to be sacrificed before day 53 posttransplantation with a median survival of 50 days posttransplantation (dpt; Fig. 5a, c). In contrast, VSV-WT-treated mice showed a 100% tumor response rate associated with rapid reduction of tumor burden. Fourteen out of 20 (70%) VSV-WT-injected tumors (two tumors per mouse) regressed completely, but unfortunately 9 out of 10 (90%) VSV-WT-treated mice developed severe neurological symptoms (e.g., hind pawn paralysis, circulation, apathy) and had to be sacrificed, leading to a median survival of 43 dpt. Only one VSV-WT-treated mouse showed long-term event-free survival. In the srVSV-treated cohort, all tumors responded and regressed over time as well (Fig. 5a). Consistently, s.c. tumors isolated 3 days posttreatment showed multiple foci of copropagation throughout the tumor diameter (Fig. 5b). But in contrast to VSV-WT, antitumor efficacy was attenuated as tumors regressed slower with first significant differences detectable at 31 dpt (*p* < 0.001). However, at 80 days posttreatment (100 dpt), 16 out of 20 tumors (80%) were eliminated and all other tumors were continuously regressing. Most importantly, all srVSV-treated mice did not show any adverse effects, so that srVSV treatment eventually led to long-term survival compared to both control cohorts (*p* < 0.0001, Fig. 5c).

### srVSV(ΔG/ΔL) exhibits reduced neurotoxicity compared to VSV-WT

**Fig. 6:**
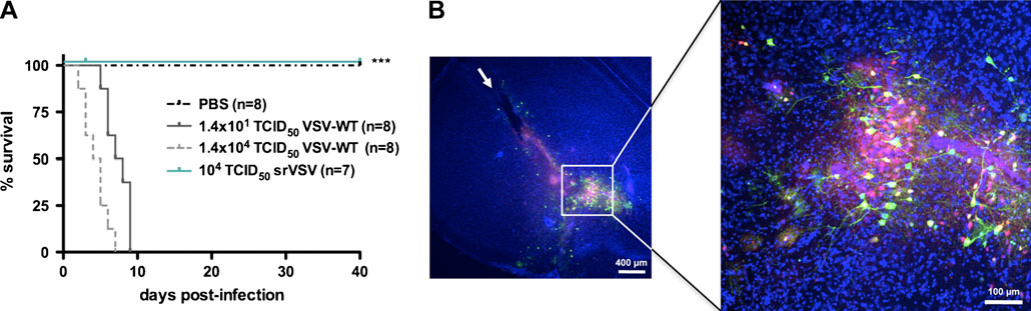
srVSV exhibits reduced neurotoxicity relative to VSV-WT. Escalating doses of 10^2^, 10^3^, and 10^4^ TCID_50_ srVSV(ΔG/ΔL) as well as 1.4 × 10^1^ and 1.4 × 10^4^ TCID_50_ VSV-WT or PBS were stereotactically injected into mouse brains. **a** Animals were monitored for signs of neurological impairment for 40 days. As all srVSV-treated cohorts showed event-free survival up to the end point, only the data set of the high-dose srVSV-treated cohort is shown. **b** Two mice of the 10^4^ TCID_50_ srVSV(ΔG/ΔL)-treated group were sacrificed at 3 dpi, brains were fixed and sections (40 μm) prepared for immunofluorescence analysis. Nuclear counterstaining was performed with TO-PRO-3 iodide. ***p < 0.001, *arrow* injection site

With srVSV(ΔG/ΔL) having shown potent antitumor activity in vitro and in vivo, its toxicity profile was evaluated after direct intracerebral administration. Mice were inoculated with either escalating doses of 10^2^ (*n* = 3), 10^3^ (*n* = 4), and 10^4^ (*n* = 7) TCID_50_ srVSV(ΔG/ΔL) or 1.4 × 10^1^ and 1.4 × 10^4^ TCID_50_ VSV-WT (each, *n* = 8) or PBS (*n* = 8), respectively. Mice were monitored for signs of neurotoxicity over a period of 40 days (Fig. 6a). Two mice of the high-dose srVSV group were sacrificed at 3 dpi, and in vivo copropagation was found to be restricted to the needle track and its proximity by immunofluorescence analysis (Fig. 6b). Most importantly, none of the srVSV(ΔG/ΔL)-injected animals showed any evidence of neuropathology with 100% of animals surviving to the end point as did the PBS-injected negative control cohort. In striking contrast, both the low- and high-dose VSV-WT cohort developed neuropathology within 2–9 dpi with all mice succumbing to neurological symptoms by day 9 and a median survival of 4.5 dpi (1.4 × 10^4^ TCID_50_ VSV-WT) and 7.5 dpi (1.4 × 10^1^ TCID_50_ VSV-WT), respectively. Hence, neurotoxicity of srVSV(ΔG/ΔL) was at least >700-fold reduced if compared to VSV-WT with differences in survival between srVSV- and VSV-WT-treated cohorts being highly significant (*p* < 0.001).

### srVSV(ΔG/ΔL) is a potent type I IFN inducer

As it was previously shown that type I IFN-inducing strains of VSV were strongly attenuated regarding their toxicity [26], the capacity of srVSV(ΔG/ΔL) to induce IFN was evaluated in murine pDC cultures. pDCs were infected with either srVSV(ΔG/ΔL), VSV*ΔG, VSVΔL-DsRed, or VSV-WT as negative and VSV*M_Q_ as positive control at an MOI of 2. Culture supernatants were collected at 24 hpi and analyzed for IFN-α via ELISA. Unsurprisingly, the VSV-M multimutated positive control VSV*M_Q_ induced the strongest type I IFN response, with 3,200.11 ± 57.02 pg IFN-α per ml supernatant, whereas VSV-WT treatment induced 18-fold lower amounts (174.47 ± 16.70 pg/ml; *p* < 0.0001), being consistent with Waibler et al. [22]. In striking contrast to VSV-WT, srVSV(ΔG/ΔL) proved to be a very potent type I IFN inducer with significantly elevated IFN-α levels of 1,035.52 ± 50.57 pg/ml (*p* < 0.0001). However, single-vector treatment with propagation-deficient VSV*ΔG and VSVΔL-DsRed induced only basal IFN-α levels.

## Discussion

Here, we developed a semireplication-competent VSV vector system composed of two separate propagation-incompetent viral vectors that shows significant anticancer activity without the neurotoxicity usually found in VSV infection of mice and nonhuman primates. Three different vector pairs of three separate propagation-incompetent vectors VSVΔP-DsRed, VSV*ΔG, and VSVΔL-DsRed were feasible, and all three combinations were able to effectively *trans*-complement each other and generate progeny virions (Figs. 1b, c and 2 and Table 1).

The combination of VSV*ΔG and VSVΔL-DsRed proved to be the most potent in terms of vector propagation (Fig. 1b, Table 1) and in vitro antitumor efficacy (Fig. 4), being only slightly attenuated compared to VSV-WT. That this combination outperforms the other srVSV systems is most likely due to the small genome size of the VSV-L gene deleted VSVΔL-DsRed vector (5.6 kB, Fig. 1a). Compared to full-length VSV, the smaller sized VSVΔL-DsRed genome is statistically favored for replication, packaging, and viral shedding. This is consistent with VSV-defective interfering particles, as apart from the 5′–3′ terminal complementarity as major determinant, genome size was also shown to impact their replicative dominance over VSV-WT [15, 27]. Moreover, the asymmetric contributions of either vector genome and particularly the overrepresentation of VSVΔL-DsRed in the srVSV(ΔG/ΔL) and the srVSV(ΔP/ΔL) system (Fig. 1c) as well as the fact that both VSVΔL-DsRed containing srVSV systems exhibit superior antitumor efficacy compared to srVSV(ΔP/ΔG) (Fig. 4) can also be explained by the genome size-dependent replicative advantage. In fact, despite the low initial MOI of 0.2, coamplification led to a nearly complete tumor cell killing in vitro at 96 hpi for glioma monolayer and spheroid cultures, underscoring the strong oncolytic potential of both srVSV(ΔG/ΔL) and srVSV(ΔP/ΔL). However, compared to srVSV(ΔP/ΔL), the (ΔG/ΔL) combination proved to be somewhat more potent in terms of vector propagation. The VSV*ΔG vector, which lacks the ability to produce infectious virus but still exhibits functional replication and transcription, provides the L polymerase for immediate VSVΔL-DsRed replication and transcription upon coinfection. This is opposed to srVSV(ΔP/ΔL), as here only double-infected cells support efficient genome replication and transcription, which might explain for the marginal reduced replication competence of the latter system. Accordingly, the most potent srVSV system, srVSV(ΔG/ΔL), was assessed for its antitumor potency in a s.c. G62 human glioblastoma xenograft model. All srVSV-treated tumors showed a clear response starting at 2 dpi. Tumors regressed and viral dissemination could be detected throughout the neoplastic tissue by immunohistochemistry (Fig. 5a, b). Although, compared to VSV-WT-treated mice tumor regression was significantly slower, srVSV treatment was not associated with any severe adverse effects (Fig. 5c). Whereas 90% of mostly (70%) tumor-free VSV-WT-treated animals had to be euthanized due to neurotoxicity, srVSV treatment resulted in long-term survival of all animals with 80% tumor clearance at 100 dpt compared to both control cohorts. Thus, it can be assumed that replication of srVSV is self-contained to the injection site and adjacent areas of the topically treated tumor, reducing the risk of a systemic infection or dissemination.

On the other hand, this intrinsic safety of binary srVSV systems may reduce therapeutic efficacy upon systemic application, as cells within target tissue need to be double infected to trigger oncolytic copropagation in the tumor. However, when delivered locoregionally, the srVSV system indeed exhibits a vastly improved therapeutic index when compared to VSV-WT, as even after direct intracerebral administration of escalating viral doses into mouse brains no toxicity could be observed. All srVSV-treated mice (low- and high-dose cohorts) showed 100% event-free survival up to the end point of the study (40 days, Fig. 6a). Again, immunohistochemistry of mice sacrificed at 3 dpi displayed locally restricted srVSV copropagation at the needle track and its close proximity (Fig. 6b). In contrast, both low- and high-dose cohorts of VSV-WT-treated mice developed neurotoxicity with a median survival of 7.5 dpi (1.4 × 10^1^ TCID_50_) or even 4.5 dpi for the high-dose cohort (1.4 × 10^4^ TCID_50_, Fig. 6a). Hence, in direct comparison with VSV-WT, the srVSV-associated neurotoxicity is at least >700-fold reduced while retaining its potent oncolytic activity.

**Fig. 7:**
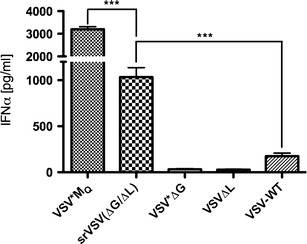
srVSV is a potent type I IFN inducer. Mouse plasmacytoid DC cultures were infected at an MOI of 2 with either srVSV(ΔG/ΔL), VSV*ΔG, VSVΔL-DsRed, VSV-WT, or the strongly type I IFN-inducing VSV*M_Q_ as a positive control (each *n* = 2). Culture supernatants were collected at 24 hpi and analyzed for IFN-α via ELISA

As infection of susceptible cells for both viral systems, VSV-WT and srVSV, is mediated by the VSV-G envelope protein, the attenuated phenotype of srVSV is not due to a shift of tropism. Instead, our data emphasize two aspects, which lead to a general and a more specific attenuation: First, the srVSV intrinsic mode of replication leads to a general attenuation per se, as copropagation is limited to foci of high vector concentrations resulting in double-infected cells and ongoing spread. At distal sites to the replicating foci, vector concentrations dramatically decrease particularly in solid tissue possibly ending off copropagation. Consistently, discrete foci of copropagation could be observed throughout the tumor diameter in intratumoral injected s.c. tumors (Fig. 5b) as well as in mouse brains after intracranial injection (Fig. 6b). Second, srVSV proved to be a very potent type I IFN inducer, inducing at least 18-fold higher IFN-α amounts when compared to VSV-WT (Fig. 7). Similar to the IFN-inducing VSV strains AV1 and AV2, we expect srVSV to be selectively attenuated in IFN-responsive, healthy cells while retaining its lytic potential in IFN-deregulated neoplastic cells. However, in comparison with AV1 and AV2, the ability to induce type I IFN is not attributed to a mutant VSV-M, which blocks host nucleocytoplasmic mRNA export, as both components of the binary system code for wild-type VSV-M [26]. In this regard we presume that due to the gene deletions, the srVSV(ΔG/ΔL) coreplication operates on a distorted transcription gradient with deficient levels of VSV-G and VSV-L particularly in the initial phase postinfection. These aberrant VSV protein levels may potentially lead to a prolonged retention time of unassembled virions within the host cell with the prolonged residence of viral RNA eventually triggering pattern recognition receptors, which can initiate a type I IFN response. The exact reason for the IFN-inducing capability of srVSV as well as its contribution to the overall attenuation of srVSV clearly requires further analysis.

In addition, the srVSV systems were also safe with respect to potential recombinatory reversion to a replication-competent phenotype, as after 20 consecutive passages at limiting dilutions, phenotypic and genotypic analyses were negative for recombinant replication-competent virus, with VSV-WT spiked positive controls validating the assays (Fig. 3). This observation is also consistent with earlier studies on the potential recombination between temperature-sensitive VSV mutants [28].

In summary, relative to VSV-WT, srVSV systems present a promising platform for virotherapeutic approaches, as they are genetically stable and exhibit considerably reduced neurotoxicity while retaining their antitumor potency. Furthermore, srVSV systems offer a strongly increased coding capacity so that both viral vectors can be “armed” to express therapeutic transgenes allowing for multipronged approaches, combining their inherent oncolytic effect with a tumor microenvironment modulating suicide and/or immunostimulatory “payload” to boost antitumor potency. Eventually, with respect to both biosafety and coding capacity, srVSV systems may not only prove valuable for oncolytic virotherapy but also represent an attractive vector vaccine platform.

## Electronic supplementary material

Below is the link to the electronic supplementary material.

ESM 1 (M4V 3,282 kb)

ESM 2 (M4V 3,453 kb)

Fig. S1 **a** Sequence identity of VSV*ΔG as well as of the de novo generated recombinants VSVΔP-DsRed and VSVΔL-DsRed was checked via VSV gene-specific reverse transcription PCR. Each individual PCR was designed to yield a different amplicon size. **b** Applied primer sets. **c** Schematic representation of the two independent qPCR primer/probe designs. *Numbers* denote the position of the respective primer/probe within the VSV-WT genome. **d** Specific sequence of the used primer/probe oligonucleotides including 5′ and 3′ modifications. *FAM* 6-carboxyfluorescein, *BHQ1* Black Hole Quencher-1, *LC610* LightCycler® Red610, *BHQ2* Black Hole Quencher-2 (GIF 106 kb)

High resolution image (EPS 10,855 kb)

Fig. S2 Details of the PCR strategy to detect reversion of deletion mutants to VSV recombinants. **a** Schematic representation of the analytical nested PCR and **b** the respective primer sets. (GIF 56 kb)

High resolution image (EPS 5,117 kb)
