# Supplementary figures and images for: Semireplication-competent vesicular stomatitis virus as a novel platform for oncolytic virotherapy
Source: J Mol Med (Berl). 2012 Jan 28;90(8):959–70. doi: 10.1007/s00109-012-0863-6 (PMC3396339; doi:10.1007/s00109-012-0863-6)

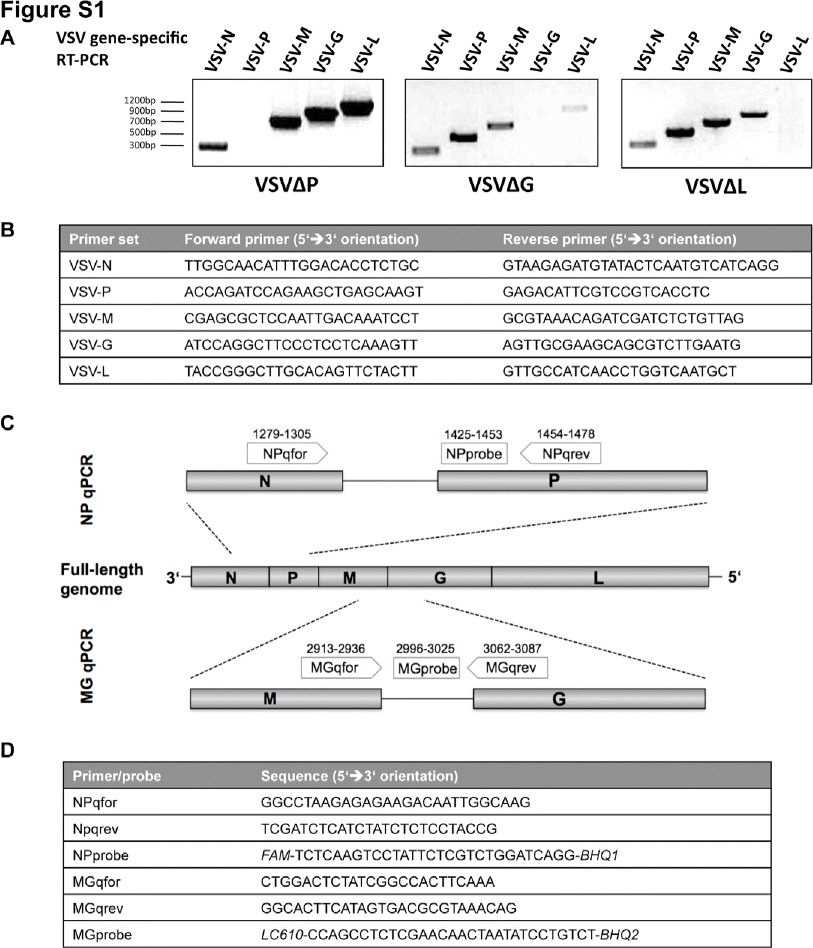

Supplement: Supplementary file 3 — a Sequence identity of VSV*ΔG as well as of the de novo generated recombinants VSVΔP-DsRed and VSVΔL-DsRed was checked via VSV gene-specific reverse transcription PCR. Each individual PCR was designed to yield a different amplicon size. b Applied primer sets. c Schematic representation of the two independent qPCR primer/probe designs. Numbers denote the position of the respective primer/probe within the VSV-WT genome. d Specific sequence of the used primer/probe oligonucleotides including 5′ and 3′ modifications. FAM 6-carboxyfluorescein, BHQ1 Black Hole Quencher-1, LC610 LightCycler® Red610, BHQ2 Black Hole Quencher-2 (GIF 106 kb) [file 109_2012_863_Fig8_ESM.gif]

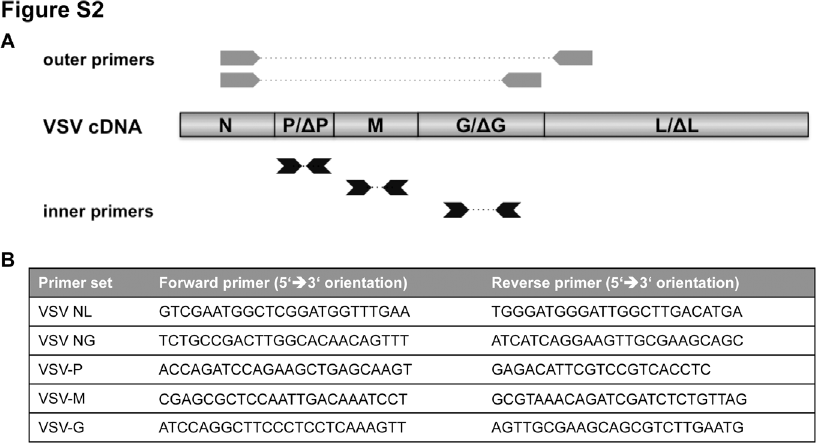

Supplement: Supplementary file 5 — Details of the PCR strategy to detect reversion of deletion mutants to VSV recombinants. a Schematic representation of the analytical nested PCR and b the respective primer sets. (GIF 56 kb) [file 109_2012_863_Fig9_ESM.gif]
